# Low hemoglobin and PSA kinetics are prognostic factors of overall survival in metastatic castration-resistant prostate cancer patients

**DOI:** 10.1038/s41598-023-29634-5

**Published:** 2023-02-15

**Authors:** Yuji Hakozaki, Yuta Yamada, Yuta Takeshima, Satoru Taguchi, Taketo Kawai, Masaki Nakamura, Takuya Iwaki, Taro Teshima, Yoshitaka Kinoshita, Yoshiyuki Akiyama, Yusuke Sato, Daisuke Yamada, Motofumi Suzuki, Haruki Kume

**Affiliations:** 1 Department of Urology, The University of Tokyo Graduate School of Medicine, 7-3-1 Hongo, Bunkyo-ku, Tokyo, 163-0033 Japan; 2 Division of Innovative Cancer Therapy, Advanced Research Center, The Institute of Medical Science, The University of Tokyo, Tokyo, Japan; 3 Department of Urology, Teikyo University Hospital, Tokyo, Japan; 4 Department of Urology, NTT Medical Center, Tokyo, Japan; 5 Department of Urology, Chiba Tokushukai Hospital, Funabashi, Chiba Japan; 6 Department of Urology, Tokyo Metropolitan Bokutoh Hospital, Tokyo, Japan

**Keywords:** Oncology, Urology

## Abstract

The objective of this study was to identify the prognostic factors and to propose a new risk model in metastatic castration-resistant prostate cancer (mCRPC) patients. The clinical data were retrospectively obtained for 102 mCRPC patients who received cancer treatment between 2005 and 2018 at the University of Tokyo Hospital. We investigated clinical and pathological parameters, including prostate-specific antigen (PSA) kinetic profiles under androgen deprivation treatment, and identified predictors of overall survival (OS). The median age and PSA were 73 (Interquartile range [IQR], 68–79) years and 5.00 (IQR, 2.77–13.6) ng/ml. The median follow-up was 34 (IQR, 17–56) months. In univariate analysis, ‘lymph node metastasis’, ‘Hemoglobin (Hb)’, ‘Time to nadir PSA (TNPSA)’, ‘PSA doubling time (PSADT)’, ‘Time to CRPC’, and ‘presence of pain’ were prognostic factors. Multivariate analysis identified ‘Hb < 11 g/dL’, ‘TNPSA < 7 months’ and ‘PSADT < 5 months’ as independent prognostic factors of OS. The high-risk group (patients with two or three factors) demonstrated shorter OS (23 vs. 50 months) with an increased risk of death (HR = 2.997; 95% CI 1.632–5.506; *P* = 0.0004). The proposed risk stratification model may contribute to the prediction of survival and provide supportive information in treatment decision-making.

## Introduction

Prostate cancer is the second most commonly diagnosed type of cancer^1^, and the number of newly diagnosed cases was estimated at 1,414,000 patients in the fiscal year 2020^2^. Prostate-specific antigen (PSA) screening increased the rates of localized-prostate cancer patients, but also detected metastatic prostate cancer in more than 10% of the screened patients^3^.

Historically, metastatic castration-sensitive prostate cancers (mCSPC) are treated with androgen deprivation therapy (ADT) by gonadotropin-releasing hormone agonists, antagonists, or orchiectomy. However, most of the ADT-treated mCSPC patients develop castration-resistant prostate cancers (CRPC) within three years^4^. CRPC is a condition in which response to ADT treatment can no longer be expected, and the entity is associated with a very poor prognosis^5^. Metastatic CRPC (mCRPC) is characterized by the rapid growth of cancer, and the median survival rate was reported as 13.2 months^6^. Although mCRPC is a lethal disease, the treatment response differs among individual cases, and risk stratification model may be useful for patient counseling at diagnosis and design of prospective trials.

In 2004, docetaxel was approved for the treatment of mCRPC patients based on the randomized control trials^7,8^. Consequently, many prognostic factors of overall survival (OS) of the mCRPC patients who received docetaxel were reported, such as visceral metastasis^9^, the number of metastatic sites^9^, presence of pain^9,10^, performance status (PS)^9,11^, progression type^9^, Gleason score (GS)^9^, PSA^9^, alkaline phosphate (ALP)^9,11^, hemoglobin (Hb)^9,11^, lactate dehydrogenase (LDH)^10^, albumin (Alb)^10^, PSA doubling time (PSADT)^11^, cycles of chemotherapy^11,12^, time to castration-resistant prostate cancer (TTCRPC)^11^.

New therapeutic options, including androgen receptor-axis targeted therapies (ARAT), were approved for mCRPC treatment in Japan based on randomized phase 3 trials in recent years: abiraterone acetate^13^, enzalutamide^14^, and cabazitaxel^15^. In mCRPC patients who received these new therapeutic agents, many prognostic factors of OS have been identified. Such factors include liver metastasis^16^, PS^16^, duration of ADT treatment^16^, time from prostate cancer diagnosis^17^, presence of pain^17^, PSA^18,19^, LDH^16,17^, Alb^16^, ALP^17^, Hb^18^, cycles of chemotherapy^18^, response to prior chemotherapy^18^, PSA decline after CRPC treatment^20,21^, age^19^, GS^19^, nadir PSA^22^, and time to nadir PSA (TNPSA)^22^.

Among these cohorts, some prognostic factors can only be obtained after CRPC treatment, such as cycles of chemotherapy, response to chemotherapy, and PSA decline after CRPC treatment^11,12,18,20,21^. Although these factors might reflect OS well, we cannot apply these factors for the OS prediction or treatment decision-making at the time of CRPC diagnosis. Therefore, we aimed to investigate the OS prognostic factors of mCRPC patients among clinical factors obtained before CRPC treatment.

## Results

### Patient characteristics

The clinical and histological features of the eligible patients are presented in Table 1. This retrospective cohort included 102 M1CRPC patients, with a median follow-up of 34 (Interquartile range [IQR], 17–56) months. The median age of patients at the CRPC diagnosis was 73 (IQR, 68–79) years. The majority (95%) of patients had metastasis at the initial diagnosis of prostate cancer, while 5 (4.9%) patients had no metastatic lesion at the time of prostate cancer diagnosis but had developed metastasis during ADT treatment. Seventy-eight out of 102 patients received combined androgen blockade therapy with anti-androgen at CRPC diagnosis. Notably, the patient characteristics based on the cutoffs of identified prognostic factors are also shown in Supplementary Table 2–4.

**Table 1:** Clinical and histological characteristics of M1CRPC patients.

	Median (IQR) or number (%)
Patients, N	102
Follow-up, months	34 (17–56)
Age at prostate cancer diagnosis, year	71 (66–78)
PSA at prostate cancer diagnosis, ng/mL	214.1 (46.5–774.3)
Clinical stage at prostate cancer diagnosis	
Tx	35 (34.3)
T1-T2	13 (12.7)
T3	39 (38.2)
T4	15 (14.7)
Nx	2 (2.0)
N0	51 (50.0)
N1	49 (48.0)
M0	5 (4.9)
M1	97 (95.1)
Extent of disease (number of bone metastases)	
1	53 (52.0)
2	19 (18.6)
3–4	22 (21.6)
No bone metastasis	8 (7.8)
Visceral metastasis	
Positive	11 (10.8)
Negative	91 (89.2)
Gleason score at prostate cancer diagnosis, n	
6	0 (0.0)
7	10 (9.8)
8	23 (22.5)
9–10	57 (55.9)
No biopsy, unknown	12 (11.8)
Localized treatment	
None	93 (91.2)
Radical prostatectomy	3 (2.9)
External beam radiation	5 (4.9)
Brachytherapy	1 (1.0)
Type of ADT	
Surgical orchiectomy	30 (29.4)
Luteinizing hormone-releasing hormone agonist	66 (64.7)
Luteinizing hormone-releasing hormone antagonist	6 (5.9)
Combined androgen blockade with antiandrogen	78 (76.5)
Presence of pain, positive	18 (17.6)
Charlson comorbidity index	
0	88 (86.3)
1–2	14 (13.7)
Nadir PSA under ADT treatment, ng/mL	0.78 (0.17–4.46)
PSA reduction rate, %	99.7 (98.2–99.9)
Time to nadir PSA from the start of ADT, months	7 (4–12)
Age at CRPC diagnosis, years	73 (68–79)
Time to CRPC from the start of ADT, months	13 (7–27)
Blood laboratory tests at CRPC diagnosis	
PSA, ng/mL	5.00 (2.77–13.6)
ALP, U/L	270 (195–374)
LDH, U/L	210 (188–238)
Hb, g/dL	12.7 (11.8–13.5)
PSA doubling time, months	2.4 (1.3–4.2)

*IQR* Interquartile range, *PSA* prostate-specific antigen, *ADT* androgen deprivation therapy, *CRPC* castration-resistant prostate cancer, *ALP* alkaline phosphatase, *LDH* Lactate dehydrogenase, *Hb* Hemoglobin.

### Treatment sequences

**Figure 1:**
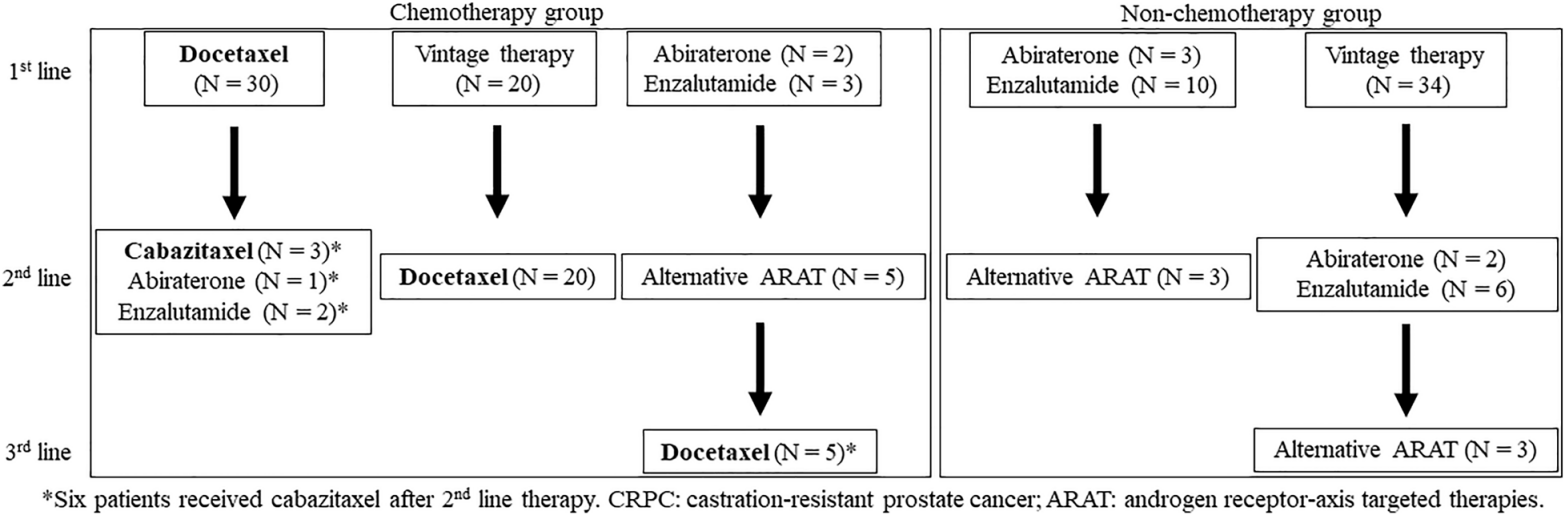
Treatment sequences of the patients in the present cohort. *CRPC* castration-resistant prostate cancer; *ARAT* androgen receptor-axis targeted therapies.

The treatment sequences for CRPC are shown in Fig. 1. Among the patients who received life-prolonging therapies, docetaxel monotherapy was the most administered regimen in the first-line treatment for CRPC (29.4%; 30 of 102 patients), followed by enzalutamide (12.7%) and abiraterone acetate (4.9%). Fifty-four (52.9%) patients received vintage therapies, including flutamide, chlormadinone, low-dose dexamethasone, or estramustine, of which 20 patients received docetaxel as a 2nd line treatment. Patients who received docetaxel as the first-line therapy received cabazitaxel or ARAT as the second-line treatment. On the other hand, patients who received ARAT as the first line received secondary ARAT agents, of which five patients received docetaxel as the third-line treatment.

### Prognostic factors for OS and risk stratification

In the univariate analysis, six prognostic factors out of 19 clinical parameters were significantly associated with the OS of mCRPC patients (Table 2). The number of factors nominated for the multivariate analysis was based on the one in ten rule (one predictive variable can be studied for every ten events.) that allowed us to include 5 factors in the analysis^23^. ‘Time to CRPC < 20 months’ was excluded in the multivariate analysis as this factor may be reflected by the combination of ‘Time to nadir PSA’ and ‘PSA doubling time’.

**Table 2:** Univariate and multivariate analysis of prognostic factors of overall survival.

	Univariate analysis		Multivariate analysis		
Factors	Hazard ratio (95%CI)	*P*-value	Hazard ratio (95%CI)	Coefficient	*P*-value
Age*^1^, year (≥ 77 vs. < 77)	0.848 (0.438–1.642)	0.6244			
PSA*^1^, ng/ml (> 800 vs. ≤ 800)	1.449 (0.781–2.689)	0.2399			
Gleason score (≥ 8 vs. < 8) (≥ 9 vs. < 9)	1.070 (0.472–2.426) 1.809 (0.968–3.381)	0.8715 0.0631			
Extent of disease*^2^ (2–4 vs. 0–1) (3–4 vs. 0–2)	1.468 (0.837–2.573) 1.068 (0.546–2.090)	0.1802 0.8484			
Lymph node metastasis (cN1 vs. cN0)	2.077 (1.149–3.752)	0.0155*	1.799 (0.929–3.484)	0.598	0.0818
Visceral metastasis (yes vs. no)	0.807 (0.287–2.273)	0.6848			
Nadir PSA, ng/mL (> 0.32 vs. ≤ 0.32)	1.584 (0.857–2.926)	0.1423			
PSA reduction rate, % (< 85.0 vs. ≥ 85.0)	1.008 (0.308–3.297)	0.9900			
Time to nadir PSA, months (< 7 vs. ≥ 7)	1.995 (1.140–3.493)	0.0156*	2.561 (1.346–4.871)	0.902	0.0041*
PSA doubling time, month (< 5 vs. ≥ 5)	3.647 (1.300–10.23)	0.0139*	3.586 (1.065–12.08)	1.097	0.0393*
Age*^3^, years (≥ 79 vs. < 79)	0.862 (0.426–1.746)	0.6805			
PSA, ng/mL (≥ 2.25 vs. < 2.25)	2.191 (0.928–5.174)	0.0735			
ALP, U/L (> 200 vs. ≤ 200)	1.043 (0.547–1.986)	0.8991			
LDH, U/L (> 190 vs. ≤ 190)	0.926 (0.467–1.834)	0.8246			
Hb, g/dL (< 11.0 vs. ≥ 11.0)	2.752 (1.247–6.074)	0.0122*	3.875 (1.661–9.044)	1.366	0.0017*
Alb, g/dL (< 4.0 vs. ≥ 4.0)	0.983 (0.452–2.138)	0.9656			
Time to CRPC, months (< 20 vs. ≥ 20)	2.040 (1.057–3.938)	0.0337*			
Presence of pain (yes vs. no)	2.255 (1.107–4.592)	0.0250*	2.074 (0.989–4.349)	0.672	0.0535
Charlson comorbidity index (0 vs. 1–2)	1.066 (0.449–2.529)	0.8850			

*CI* confidence interval; *PSA* prostate-specific antigen; *ALP* alkaline phosphatase; *LDH* lactate dehydrogenase; *Hb* Hemoglobin; *Alb* Albumin; *CRPC* castration-resistant prostate cancer.

*Statistically significant with a *P* < 0.05.

*^1^At prostate cancer diagnosis.

*^2^Extent of disease is defined by a number of bone metastases.

*^3^At CRPC diagnosis.

**Figure 2:**
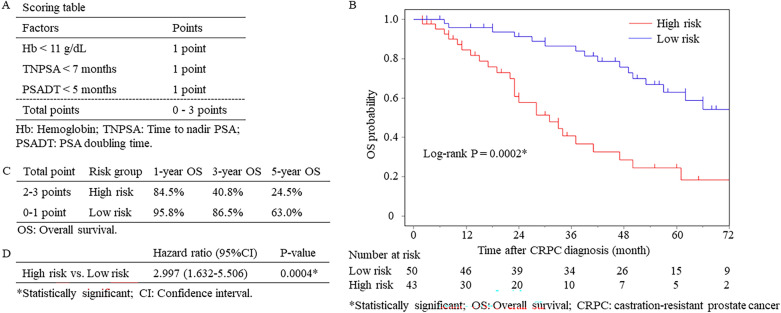
Kaplan–Meier curves according to the risk stratification (**A**) Scoring table for risk stratification (**B**) Kaplan Meier plot of overall survival stratified by risk groups (**C**) Overall survival according to the risk groups. (**D**) A hazard ratio of patients in the high-risk group.

**Figure 3:**
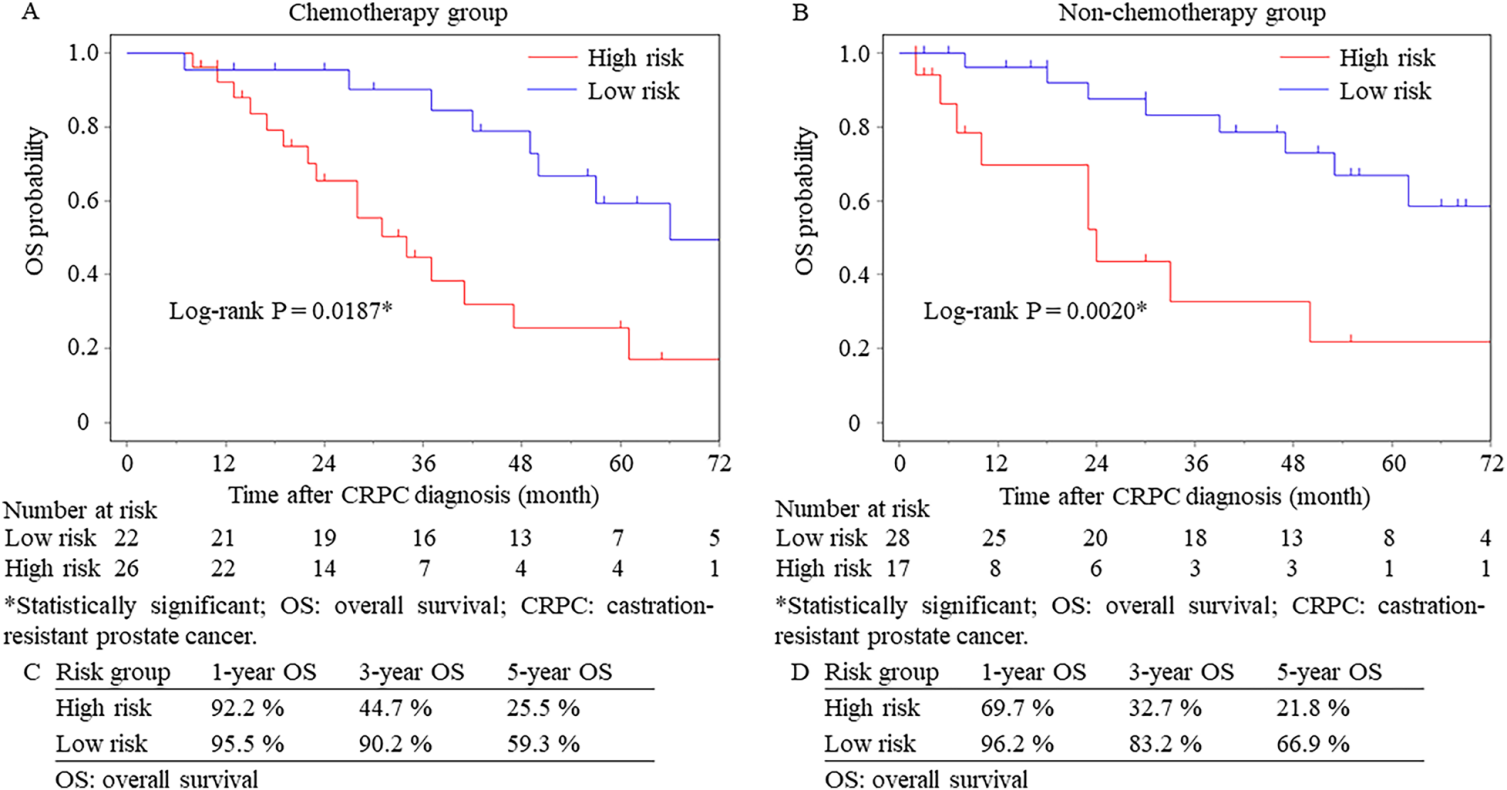
Kaplan–Meier curves for the patients (**A**) with and (**B**) without chemotherapy treatment. The overall survival rates for the patients treated (**C**) with and (**D**) without chemotherapy treatment.

Multivariate analysis further identified ‘Hb < 11 g/dL’, ‘TNPSA < 7 months’ and ‘PSADT < 5 months’ as independent predictors of OS. Risk stratification was performed based on these predictors (Fig. 2A). Patients with two or three factors were classified as the high-risk group, and patients with zero or one risk factor were classified as the low-risk group. The median OS was 23 months (95% Confidence interval [CI], 20 to 38 months) in the high-risk group and 50 months (95% CI 41 to 58 months) in the low-risk group, respectively (Fig. 2B). In addition, 1-year, 3-year, and 5-year survival rates in the high-risk and low-risk groups were 84.5% vs. 95.8%, 40.8% vs. 86.5%, and 24.5% vs. 63.0%, respectively (Fig. 2C). Patients in the high-risk group demonstrated an increased risk of death (HR = 2.997; 95% CI 1.632–5.506; *P* = 0.0004) compared to the patients in the low-risk group (Fig. 2D). Internal validation using a bootstrap method showed a C-index of 0.69, 0.66, 0.67 for the proposed risk models based on the entire cohort, chemotherapy group, and non-chemotherapy group, respectively (Figs. 2B, 3A,B).

A nomogram was developed for predicting 3-year OS based on the prognostic factors (Supplementary Fig. 1A). The calibration plots demonstrated good consistency between predicted and actual 3-year OS (Supplementary Fig. 1B).

### Subgroup analysis

Due to the treatment variability in this cohort, patients were divided into groups according to the use of chemotherapy at any time during the treatment (Fig. 1). We further conducted survival analysis on these two groups to ascertain whether the risk classification would be applicable regardless of chemotherapy treatment.

Overall, 56 (54.9%) patients received chemotherapy (chemotherapy group), and 46 (45.1%) patients received ARAT or vintage therapies (non-chemotherapy group) (Supplementary Table 1). Between chemotherapy and non-chemotherapy group, age at prostate cancer and CRPC diagnosis, TTCRPC, and PSA reduction rate (PSARR), the value of PSA and Hb were statistically different.

Patients were categorized into two risk groups based on the total points that were calculated by the number of risk factors (Supplementary Table 5). After subgrouping, a statistically significant difference in OS was observed between risk groups for patients regardless of chemotherapy treatment (Fig. 3A,B). The 3-year survival rates for the patients with and without chemotherapy treatment were 90.2% and 83.2% in the low-risk group and 44.7% and 32.7% in the high-risk group, respectively (Fig. 3C,D). Multivariate analysis was performed using the three prognostic factors for the subgroups divided by the presence of chemotherapy use (Supplementary Table 6). ‘Hb < 11 g/dL’ was statistically significant in the non-chemotherapy group, while ‘TNPSA < 7 months’ and ‘PSA doubling time < 5 months’ were significant prognostic factors of OS in the chemotherapy group.

## Discussion

We identified Hb < 11 g/dL, TNPSA < 7 months and PSADT < 5 months as independent prognostic factors in mCRPC patients. Based on our new risk model, patients with a higher risk showed worse survival rates regardless of the use of chemotherapy treatment. Additionally, the nomogram that was developed in the present study showed optimal agreement between predicted and actual observation regarding 3-year OS.

Our cohort defined OS as the time from CRPC diagnosis until death, though other mCRPC cohorts calculated OS from the time point of the treatment randomization^9,16^ or treatment initiation^17,20^. Several cohorts assessed OS prediction factors by the same OS definition^19,22^ as the present study and demonstrated that age^19^, GS^19^, PSA^19^, nadir PSA^22^, and TNPSA^22^ were independent OS prognostic factors of mCRPC patients. We identified ‘Hb < 11 g/dL’ as a new prognostic factor of OS in non-chemotherapy treated patients. Notably, this factor was not assessed in the previous cohorts^19,22^.

Several CRPC cohorts reported a low Hb level as an OS prognostic factor, both for patients receiving any treatment^9^, and for patients who received chemotherapy^11,18^. However, a subanalysis of the impact of low Hb levels in patients who did not receive chemotherapy has not been conducted previously. Given this background, we further divided patients according to the presence of chemotherapy treatment and identified ‘Hb < 11 g/dL’ as a prognostic factor of the patients who never received chemotherapy treatment as well. Anemia worsens the prognosis of patients in other cancer patient cohorts^24,25^. Our cohort consists of the patients diagnosed as CRPC after ADT treatment for metastatic prostate cancer treatment, and these patients were assumed to be in chronic and progressive disease status. Several factors might induce anemia, such as bone marrow replacement by metastatic cells^26^, chronic inflammation^27^, and cytokine-mediated disorder^28^. The low Hb level might reflect these factors and impact the OS consequently.

In the present study, ‘PSADT < 5 months’ was a prognostic factor of OS in mCRPC patients. PSADT was previously reported as a predictor of OS in nonmetastatic CRPC^29^. In mCRPC patients, shorter PSADT increased the risk of overall mortality in the TAX327 cohort^9^. However, the PSADT used in the study was basically calculated at the point of treatment initiation, in which CRPC treatment was initiated after a PSA level of 100 ng/mL. In our cohort, PSADT predicted OS using much lower PSA levels measured at the time of CRPC diagnosis.

In this cohort, the cut-off value of PSADT was five months, which was longer than the 46.3 days that was previously reported in the CRPC cohort with docetaxel treatment by Qu et al.^11^. Suggested cut-off values vary greatly among publications depending on the method of calculation, mainly because of the difference in time-points of PSA measurement. Qu Y.Y. et al. calculated PSADT using two PSA values at the time of CRPC diagnosis and the most recent PSA level before the CRPC diagnosis^11^. By contrast, we used all PSA values within 12 months according to the recommendation of PSA Working Group^30^. However, the disadvantage of this method is that the calculation becomes complicated when using three or more PSA values.

Our cohort has several limitations. First, clinical data were retrieved retrospectively. To confirm the utility of the identified prognostic factors, prospective studies are necessary. Second, several cohorts showed PS as a prognostic factor of OS^9,11,16^. However, this cohort did not assess PS due to missing data. Third, treatment strategy was not consistent in this cohort, so while this study may reflect real-world clinical situations, there may be a bias concerning the selection of treatment. Fourth, as many as 45% of the patients in this study have never received chemotherapy, due to mental and physical intolerance to chemotherapy. Similar results were observed in a previous study in which 61% of the patients received docetaxel among the mCRPC patients diagnosed between 2010–2013^31^. Another study by George et al. reported that only 8% of the patients received docetaxel treatment as 2nd line treatment among the patients who received abiraterone or enzalutamide as 1st line. Fifth, the sample size of this study was limited and patients were treated with various types of treatment. Therefore, future studies with external validation may be required to determine the generalizability of the risk stratification that was presented in this study.

Despite these limitations, our analysis showed the impact of TNPSA, PSADT, and Hb on the prognosis of mCRPC patients. Since the factors analyzed were limited to those which could be generated at the time of mCRPC, this risk stratification may be used to predict the prognosis of mCRPC patients during routine patient treatment. This finding requires further validation by prospective cohorts.

## Materials and methods

### Study design

This retrospective cohort included patients diagnosed as mCRPC between 2005 and 2018 at the University of Tokyo Hospital. This study was performed according to the provisions of the Declaration of Helsinki and approved by the ‘Ethics Committee of the Tokyo University Hospital’ (approval number 3124). Regarding the present study, the ‘Ethics Committee of the Tokyo University Hospital’ waived the requirement of the written informed consent.

### Patient selection

Clinical data of patients that showed metastatic lesions at CRPC diagnosis were retrospectively extracted from medical records at the University of Tokyo Hospital. Metastatic lesions were identified by conventional imaging techniques such as bone scans and computed tomography of the chest, abdomen, and pelvis. CRPC was defined as having PSA or radiographic progression under castrated status. ‘Castrated status’ was defined as ‘serum total testosterone < 50 ng/dL’ or ‘condition in which ADT treatment is performed either by surgical orchiectomy or luteinizing hormone-releasing agonist/antagonist’^32^. PSA progression was defined as an increase in PSA level of ≥ 25% and ≥ 2 ng/mL above the nadir^32^. Radiographic progression was defined as two or more new metastatic lesions detected on the bone scans^33^, or one lesion detected and confirmed as adenocarcinoma by biopsy of the metastatic lesion.

A total of 13 patients were excluded due to insufficient information. Two patients were considered intolerable for CRPC treatment due to poor general condition, and were excluded for the present study. Two patients had other concomitant aggressive malignancies and were excluded because of inability to evaluate metastatic lesions. In total, 102 patients were selected for the final analysis.

### Data retrieval

Clinical parameters were reviewed from the clinical records, including age, prostate biopsy GS before treatment, presence of lymph node metastasis, medications, complications, blood tests at the time of CRPC diagnosis, the use of local therapy, and the treatment sequences for CRPC. The extent of disease was determined by the radiographic image by bone scans^34^. TTCRPC was defined as the duration between the start of ADT treatment and CRPC diagnosis. The presence of symptomatic pain was decided by whether analgesic agents were prescribed at the time of CRPC diagnosis. Charlson comorbidity index was calculated according to the definition developed by Charlson et al.^35^. All PSA results obtained during a 12 month time span leading up to CRPC diagnosis were collected, and used for the PSADT calculation by the web-based calculator from Memorial Sloan Kettering Cancer Center (https://www.mskcc.org/nomograms/prostate/psa_doubling_time). PSA measurements under 0.2 ng/mL were not used in the calculation of PSADT^30^. PSARR was calculated using 1 − (nadir PSA)/(PSA value at prostate cancer diagnosis). TNPSA was defined as the time interval between ADT initiation and nadir PSA during ADT treatment before CRPC diagnosis. OS was defined as the time from CRPC diagnosis until death from any cause or the last follow-up.

### Statistical analysis

Clinical data were shown by the frequency and the percentage of categorical variables, or the median value attached with the IQR for continuous variables. Regarding continuous variables, the cutoff values were determined by the closest point to the upper left corner of the ROC curve analysis (Youden index) and rounded to the clinically significant value. Univariate and multivariate analyses were performed using Cox proportional hazards regression model to identify independent factors predicting OS. Associations between continuous variables were compared by the Mann–Whitney U test, and the difference of categorical values was compared by Fisher’s exact test or Pearson’s chi-square test. All *P* values with < 0.05 were considered statistically significant. Statistically significant prognostic factors in the univariate analysis were included in the multivariate analysis. Patients were categorized into high and low-risk groups based on the aforementioned identified factors. The Kaplan–Meier curves of OS in each risk group were analyzed by log-rank test to evaluate the difference in OS among risk groups. The internal validation was carried out by calculating C-index of the risk model based on 1,000 bootstrap samples. A nomogram was developed using a Cox regression model based on the independent prognostic factors identified in the multivariate analysis. Calibration curves of the nomogram for 3-year OS were generated to determine the deviation of the observed probability from the predicted probability of survival using 200 bootstrap resamples. JMP 16.1.0 (SAS Institute Inc., Cary, NC, USA) was used for statistical analysis except for the calculation of the C-index, development of the nomogram, and construction of the calibration curves in which R Version 3.6.0 (Comprehensive R Archive Network) was used.

## Supplementary Information

Supplementary Legends.

Supplementary Figure 1.

Supplementary Tables.

## Data Availability

The dataset used in the present study is not publicly available due to the ongoing clinical studies based on the same dataset. However, it can be used by a reasonable request to the corresponding author.
