# Supplementary material for: Low hemoglobin and PSA kinetics are prognostic factors of overall survival in metastatic castration-resistant prostate cancer patients
Source: Sci Rep. 2023 Feb 15;13:2672. doi: 10.1038/s41598-023-29634-5 (PMC9931698; doi:10.1038/s41598-023-29634-5)
Supplement: Supplementary file 1 — Supplementary Legends. [file 41598_2023_29634_MOESM1_ESM.docx]

**Supplementary Figure 1** Nomogram for overall survival of the patients with metastatic castration resistant prostate cancer. (A) nomogram predicting 3-year survival based on the identified prognostic factors. (B) Calibration plots between observed and predicted frequencies for 3-year survival.

**Supplementary Table 1** Clinical and histological characteristics of patients according to the chemotherapy treatment

**Supplementary Table 2** Clinical and histological characteristics of patients according to the value of time to nadir PSA

**Supplementary Table 3** Clinical and histological characteristics of patients according to the value of PSA doubling time

**Supplementary Table 4** Clinical and histological characteristics of patients according to the value of hemoglobin

**Supplementary Table 5** Hazard ratios according to the number of identified prognostic factors of overall survival

**Supplementary Table 6** Multivariate analysis of identified prognostic factors of overall survival in the chemotherapy and non-chemotherapy group
