# Supplementary figures and images for: Low hemoglobin and PSA kinetics are prognostic factors of overall survival in metastatic castration-resistant prostate cancer patients
Source: Sci Rep. 2023 Feb 15;13:2672. doi: 10.1038/s41598-023-29634-5 (PMC9931698; doi:10.1038/s41598-023-29634-5)

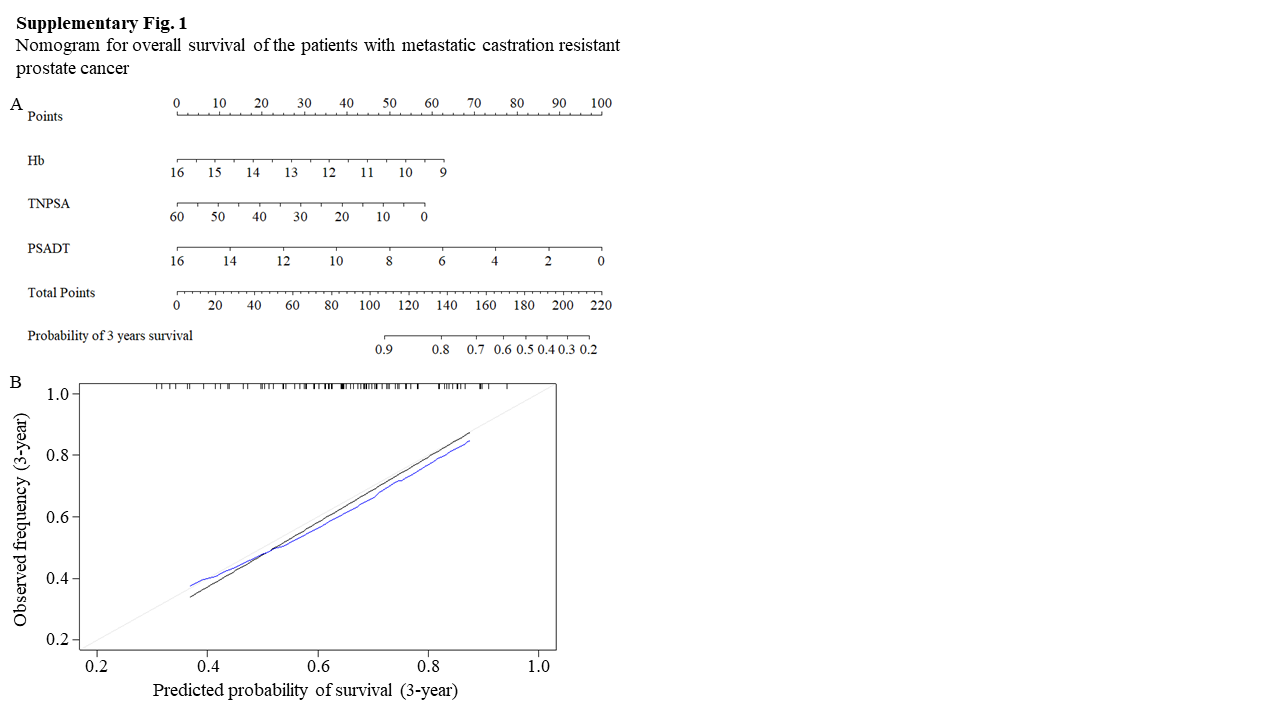

Supplement: Supplementary file 2 — Supplementary Figure 1. [file 41598_2023_29634_MOESM2_ESM.tif]
