# Supplementary material for: Low hemoglobin and PSA kinetics are prognostic factors of overall survival in metastatic castration-resistant prostate cancer patients
Source: Sci Rep. 2023 Feb 15;13:2672. doi: 10.1038/s41598-023-29634-5 (PMC9931698; doi:10.1038/s41598-023-29634-5)
Supplement: Supplementary file 3 — Supplementary Tables. [file 41598_2023_29634_MOESM3_ESM.docx]

Low hemoglobin and PSA kinetics are prognostic factors of overall survival in metastatic castration-resistant prostate cancer patients

Yuji Hakozaki, Yuta Yamada, Yuta Takeshima, Satoru Taguchi, Taketo Kawai, Masaki Nakamura, Takuya Iwaki, Taro Teshima, Yoshitaka Kinoshita, Yoshiyuki Akiyama, Yusuke Sato, Daisuke Yamada, Motofumi Suzuki, Haruki Kume

**Supplementary Table 1** Clinical and histological characteristics of patients according to the chemotherapy treatment

|  | Non-chemotherapy group | Chemotherapy group | P-value |
| --- | --- | --- | --- |
|  | Median (IQR) or number (%) | |  |
| Patients, N | 46 | 56 |  |
| Follow-up, months | 32 (9.5-55) | 34 (19-58) | 0.311 |
| Age at prostate cancer diagnosis, year | 76 (68-79) | 68 (63-74) | < 0.001* |
| PSA at prostate cancer diagnosis, ng/mL | 220 (61.9-775) | 189.1 (32.5-759) | 0.503 |
| Clinical stage at prostate cancer diagnosis  Tx  T1-T2  T3  T4  Nx  N0  N1  M0  M1 | 14 (30.4)  7 (15.2)  20 (43.5)  5 (10.9)  2 (4.3)  25 (54.3)  19 (41.3)  1 (2.1)  45 (97.8) | 21 (37.5)  6 (10.7)  19 (33.9)  10 (17.9)  0 (0.0)  26 (46.4)  30 (53.6)  4 (7.14)  52 (92.9) | 0.441    0.302  0.248 |
| Extent of disease  1  2  3-4  No bone metastasis | 23 (50.0)  12 (26.1)  9 (19.6)  2 (4.4) | 30 (53.6)  7 (12.5)  13 (23.2)  6 (10.7) | 0.288 |
| Visceral metastasis  Positive  Negative | 4 (8.7)  42 (91.3) | 7 (12.5)  49 (87.5) | 0.538 |
| Gleason score at prostate cancer diagnosis, n  6  7  8  9-10  No biopsy, unknown | 0 (0.0)  4 (8.7)  11 (23.9)  24 (52.2)  7 (15.2) | 0 (0.0)  6 (10.7)  12 (21.4)  33 (58.9)  5 (8.9) | 0.874 |
| Localized treatment  None  Radical prostatectomy  External beam radiation  Brachytherapy | 43 (93.5)  1 (2.2)  2 (4.3)  0 (0.0) | 50 (89.3)  2 (3.6)  3 (5.4)  1 (1.8) | 1.000 |
| Presence of pain, positive | 8 (17.4) | 10 (17.9) | 0.951 |
| Charlson comorbidity index  0  1-2 | 42 (91.3)  4 (8.7) | 46 (82.1)  10 (17.9) | 0.250 |
| Nadir PSA under ADT treatment, ng/mL | 0.58 (0.11-2.53) | 1.17 (0.23-9.18) | 0.0503 |
| PSA reduction rate, % | 99.8 (98.6-100) | 99.5 (95.1-99.9) | 0.036* |
| Time to nadir PSA from the start of ADT, months | 7 (4.75-13) | 6 (3-10) | 0.108 |
| Age at CRPC diagnosis, years | 78 (72-82) | 70 (65-76) | < 0.001* |
| Time to CRPC from the start of ADT, months | 19 (9-32) | 10 (7-18) | 0.015* |
| Blood laboratory tests at CRPC diagnosis  PSA, ng/mL  ALP, U/L  LDH, U/L  Hb, g/dL | 3.92 (2.62-8.27)  277 (192-345)  210 (187-237)  12.6 (11.3-13.5) | 6.13 (3.10-17.7)  270 (196-402)  207 (188-244)  13.0 (12.3-13.6) | 0.038*  0.818  0.837  0.023* |
| PSA doubling time, months | 2.6 (1.6-4.5) | 1.8 (1.2-4.1) | 0.109 |

IQR: Interquartile range, PSA: prostate-specific antigen, ADT: androgen deprivation therapy, CRPC: castration-resistant prostate cancer, ALP: alkaline phosphatase, LDH: Lactate dehydrogenase, Hb: Hemoglobin, *Statistically significant with a P < 0.05

**Supplementary Table 2** Clinical and histological characteristics of patients according to the value of time to nadir PSA

|  | Time to nadir PSA < 7 months | Time to nadir PSA ≥ 7 months | P-value |
| --- | --- | --- | --- |
|  | Median (IQR) or number (%) | |  |
| Patients, N | 49 | 53 |  |
| Follow-up, months | 23 (11-43) | 47 (29-62) | < 0.001* |
| Age at prostate cancer diagnosis, year | 71 (68-77) | 71 (64-78) | 0.933 |
| PSA at prostate cancer diagnosis, ng/mL | 215 (51-712) | 139 (46-886) | 0.828 |
| Clinical stage at prostate cancer diagnosis  Tx  T1-T2  T3  T4  Nx  N0  N1  M0  M1 | 16 (32.7)  6 (12.2)  16 (32.7)  11 (22.4)  0 (0.0)  24 (49.0)  25 (51.0)  2 (4.1)  47 (95.9) | 19 (35.8)  7 (13.2)  23 (43.4)  4 (7.5)  2 (3.8)  27 (50.9)  24 (45.3)  3 (5.7)  50 (94.3) | 0.101    0.692  0.712 |
| Extent of disease  1  2  3-4  No bone metastasis | 23 (46.9)  10 (20.4)  14 (28.6)  2 (4.1) | 30 (56.6)  9 (17.0)  8 (15.1)  6 (11.3) | 0.229 |
| Visceral metastasis  Positive  Negative | 6 (12.2)  43 (87.8) | 5 (9.4)  48 (90.6) | 0.648 |
| Gleason score at prostate cancer diagnosis, n  6  7  8  9-10  No biopsy, unknown | 0 (0.0)  4 (8.2)  7 (14.3)  31 (63.3)  7 (14.3) | 0 (0.0)  6 (11.3)  16 (30.2)  26 (49.1)  5 (9.4) | 0.137 |
| Localized treatment  None  Radical prostatectomy  External beam radiation  Brachytherapy | 44 (89.8)  2 (4.1)  2 (4.1)  1 (2.0) | 49 (92.5)  1 (1.9)  3 (5.7)  0 (0.0) | 0.744 |
| Presence of pain, positive | 10 (20.4) | 8 (15.1) | 0.482 |
| Charlson comorbidity index  0  1-2 | 42 (85.7)  7 (14.3) | 46 (86.8)  7 (13.2) | 0.874 |
| Nadir PSA under ADT treatment, ng/mL | 1.57 (0.36-10.6) | 0.38 (0.10-2.63) | 0.006* |
| PSA reduction rate, % | 99.1 (95.8-99.8) | 99.8 (99.1-99.8) | 0.002* |
| Time to nadir PSA from the start of ADT, months | 4 (3-5) | 12 (8-17) | < 0.001* |
| Age at CRPC diagnosis, years | 73 (68-78) | 75 (68-81) | 0.282 |
| Time to CRPC from the start of ADT, months | 7 (5-10) | 19 (14-50) | < 0.001* |
| Blood laboratory tests at CRPC diagnosis  PSA, ng/mL  ALP, U/L  LDH, U/L  Hb, g/dL | 7.87 (4.14-17.5)  279 (198-515)  203 (182-241)  12.8 (11.7-13.5) | 3.50 (2.30-7.80)  263 (179-341)  213 (195-238)  12.7 (12.1-13.6) | < 0.001*  0.162  0.538  0.822 |
| PSA doubling time, months | 1.6 (0.9-2.5) | 3.6 (2.0-3.6) | < 0.001* |

IQR: Interquartile range, PSA: prostate-specific antigen, ADT: androgen deprivation therapy, CRPC: castration-resistant prostate cancer, ALP: alkaline phosphatase, LDH: Lactate dehydrogenase, Hb: Hemoglobin, *Statistically significant with a P < 0.05

**Supplementary Table 3** Clinical and histological characteristics of patients according to the value of PSA doubling time

|  | PSA doubling time < 5 months | PSA doubling time ≥ 5 months | P-value |
| --- | --- | --- | --- |
|  | Median (IQR) or number (%) | |  |
| Patients, N | 81 | 17 |  |
| Follow-up, months | 30 (14-52) | 56 (17-78) | 0.011* |
| Age at prostate cancer diagnosis, year | 71 (65-77) | 75 (67-81) | 0.290 |
| PSA at prostate cancer diagnosis, ng/mL | 217 (46.0-791) | 139 (54.7-586) | 0.666 |
| Clinical stage at prostate cancer diagnosis  Tx  T1-T2  T3  T4  Nx  N0  N1  M0  M1 | 29 (35.8)  7 (8.6)  33 (40.7)  12 (14.8)  2 (2.5)  39 (48.1)  40 (49.4)  4 (4.9)  77 (95.1) | 5 (29.4)  6 (35.3)  4 (23.5)  2 (11.8)  0 (0.0)  1 (5.9)  16 (94.1)  1 (5.9)  16 (94.1) | 0.019*    0.251  0.872 |
| Extent of disease  1  2  3-4  No bone metastasis | 41 (50.6)  15 (18.5)  20 (24.7)  5 (6.2) | 11 (64.7)  3 (17.7)  1 (5.9)  2 (11.8) | 0.325 |
| Visceral metastasis  Positive  Negative | 10 (12.4)  71 (87.7) | 1 (5.9)  16 (94.1) | 0.443 |
| Gleason score at prostate cancer diagnosis, n  6  7  8  9-10  No biopsy, unknown | 0 (0.0)  5 (6.2)  16 (19.8)  49 (60.5)  11 (13.6) | 0 (0.0)  4 (23.5)  6 (35.3)  6 (35.3)  1 (5.9) | 0.026* |
| Localized treatment  None  Radical prostatectomy  External beam radiation  Brachytherapy | 75 (92.6)  2 (2.5)  4 (4.9)  0 (0.0) | 14 (82.4)  1 (5.9)  1 (5.9)  1 (5.9) | 0.186 |
| Presence of pain, positive | 18 (22.2) | 0 (0.0) | 0.032* |
| Charlson comorbidity index  0  1-2 | 69 (85.2)  12 (14.8) | 16 (94.1)  1 (5.9) | 0.324 |
| Nadir PSA under ADT treatment, ng/mL | 0.83 (0.23-4.85) | 0.20 (0.01-1.55) | 0.019* |
| PSA reduction rate, % | 99.6 (98.0-99.9) | 99.9 (98.8-100) | 0.044* |
| Time to nadir PSA from the start of ADT, months | 6 (4-10) | 13 (3-19) | 0.045* |
| Age at CRPC diagnosis, years | 73 (68-78) | 77 (71-84) | 0.015* |
| Time to CRPC from the start of ADT, months | 10 (7-19) | 45 (17-96) | < 0.001* |
| Blood laboratory tests at CRPC diagnosis  PSA, ng/mL  ALP, U/L  LDH, U/L  Hb, g/dL | 5.40 (3.10-14.9)  281 (194-454)  210 (188-237)  12.8 (11.8-13.6) | 3.02 (2.14-8.80)  217 (179-322)  205 (180-237)  12.6 (11.9-13.4) | 0.023*  0.096  0.707  0.538 |
| PSA doubling time, months | 1.8 (1.2-3.2) | 7.3 (6.2-9.7) | < 0.001* |

IQR: Interquartile range, PSA: prostate-specific antigen, ADT: androgen deprivation therapy, CRPC: castration-resistant prostate cancer, ALP: alkaline phosphatase, LDH: Lactate dehydrogenase, Hb: Hemoglobin, *Statistically significant with a P < 0.05

**Supplementary Table 4** Clinical and histological characteristics of patients according to the value of hemoglobin

|  | Hb < 11 g/dL | Hb ≥ 11 g/dL | P-value |
| --- | --- | --- | --- |
|  | Median (IQR) or number (%) | |  |
| Patients, N | 11 | 86 |  |
| Follow-up, months | 18 (7.0-33) | 40 (20-57) | 0.011* |
| Age at prostate cancer diagnosis, year | 74 (68-81) | 72 (65-77) | 0.102 |
| PSA at prostate cancer diagnosis, ng/mL | 594 (75.2-2340) | 189 (36.8-692) | 0.130 |
| Clinical stage at prostate cancer diagnosis  Tx  T1-T2  T3  T4  Nx  N0  N1  M0  M1 | 4 (36.4)  2 (18.2)  5 (45.5)  0 (0.0)  0 (0.0)  6 (54.5)  5 (45.5)  1 (9.1)  10 (90.9) | 29 (33.7)  11 (12.8)  33 (38.4)  13 (15.1)  2 (2.3)  45 (52.3)  39 (45.3)  4 (4.7)  82 (95.4) | 0.358    0.951  0.531 |
| Extent of disease  1  2  3-4  No bone metastasis | 1 (9.1)  4 (36.4)  4 (36.4)  2 (18.2) | 48 (55.8)  15 (17.4)  17 (19.8)  6 (7.0) | 0.010* |
| Visceral metastasis  Positive  Negative | 3 (27.3)  8 (72.7) | 8 (9.3)  78 (90.7) | 0.077 |
| Gleason score at prostate cancer diagnosis, n  6  7  8  9-10  No biopsy, unknown | 0 (0.0)  1 (9.1)  1 (9.1)  7 (63.6)  2 (18.2) | 0 (0.0)  7 (8.1)  21 (24.4)  48 (55.8)  10 (11.6) | 0.544 |
| Localized treatment  None  Radical prostatectomy  External beam radiation  Brachytherapy | 11 (100)  0 (0.0)  0 (0.0)  0 (0.0) | 77 (89.5)  3 (3.5)  5 (5.8)  1 (1.2) | 1.000 |
| Presence of pain, positive | 3 (27.3) | 14 (16.3) | 0.367 |
| Charlson comorbidity index  0  1-2 | 8 (72.7)  3 (27.3) | 75 (87.2)  11 (12.8) | 0.198 |
| Nadir PSA under ADT treatment, ng/mL | 1.30 (0.38-15.9) | 0.75 (0.14-4.16) | 0.189 |
| PSA reduction rate, % | 99.1 (95.6-100) | 99.7 (98.3-99.9) | 0.759 |
| Time to nadir PSA from the start of ADT, months | 5 (3-15) | 7 (4-12) | 0.711 |
| Age at CRPC diagnosis, years | 75 (69-84) | 74 (68-79) | 0.196 |
| Time to CRPC from the start of ADT, months | 9 (5-27) | 14 (8-29) | 0.210 |
| Blood laboratory tests at CRPC diagnosis  PSA, ng/mL  ALP, U/L  LDH, U/L  Hb, g/dL | 23.1 (2.48-8.25)  381 (195-1260)  206 (183-259)  10.1 (9.9-10.7) | 4.60 (2.77-12.9)  267 (194-346)  210 (188-238)  12.9 (12.3-13.6) | 0.467  0.057  0.856  < 0.001* |
| PSA doubling time, months | 1.5 (1.3-2.5) | 2.6 (1.4-4.2) | 0.412 |

IQR: Interquartile range, PSA: prostate-specific antigen, ADT: androgen deprivation therapy, CRPC: castration-resistant prostate cancer, ALP: alkaline phosphatase, LDH: Lactate dehydrogenase, Hb: Hemoglobin, *Statistically significant with a P < 0.05

**Supplementary Table 5** Hazard ratios according to the number of identified prognostic factors of overall survival

| Risk group | Total points | No. of patients (%) | Hazard ratio (95% CI) | P-value |
| --- | --- | --- | --- | --- |
| Low risk | 0 point | 10 (10.8) | - | - |
|  | 1 point | 40 (43.0) | 4.910 (0.654-36.85) | 0.1218 |
| High risk | 2 points | 37 (39.8) | 10.54 (1.405-79.07) | 0.0220* |
|  | 3 points | 6 (6.5) | 105.6 (11.40-979.0) | < 0.0001* |

CI: confidence interval; *Statistically significant with a P < 0.05. The hazard ratio was calculated relative to the patients with 0 point.

**Supplementary Table 6** Multivariate analysis of identified prognostic factors of overall survival in the chemotherapy and non-chemotherapy group

|  | Non-chemotherapy group | |  | Chemotherapy group | |
| --- | --- | --- | --- | --- | --- |
|  | Multivariate analysis | |  | Multivariate analysis |  |
| Factors | Hazard ratio (95%CI) | P-value |  | Hazard ratio (95%CI) | P-value |
| Time to nadir PSA, months (< 7 vs. ≥ 7) | 2.019 (0.758-5.372) | 0.1596 |  | 2.486 (1.050-5.881) | 0.0383* |
| PSA doubling time, month (< 5 vs. ≥ 5) | 5.370 (0.703-41.00) | 0.1051 |  | 7.139 (1.450-35.14) | 0.0156* |
| Hb, g/dL (< 11.0 vs. ≥ 11.0) | 8.830 (2.685-29.04) | 0.0003* |  | 0.607 (0.105-3.154) | 0.5776 |

CI: confidence interval; PSA: prostate-specific antigen; Hb: Hemoglobin; *Statistically significant with a P < 0.05
